# Halogen Bonding in Haspin-Halogenated Tubercidin Complexes: Molecular Dynamics and Quantum Chemical Calculations

**DOI:** 10.3390/molecules27030706

**Published:** 2022-01-21

**Authors:** Yujing Zhou, Ming Wah Wong

**Affiliations:** Department of Chemistry, National University of Singapore, 3 Science Drive 3, Singapore 117543, Singapore; chmyuji@nus.edu.sg

**Keywords:** halogen bond, noncovalent interaction, molecular dynamics simulation, density functional theory (DFT), drug–ligand interaction

## Abstract

Haspin, an atypical serine/threonine protein kinase, is a potential target for cancer therapy. 5-iodotubercidin (5-iTU), an adenosine derivative, has been identified as a potent Haspin inhibitor in vitro. In this paper, quantum chemical calculations and molecular dynamics (MD) simulations were employed to identify and quantitatively confirm the presence of halogen bonding (XB), specifically halogen∙∙∙π (aromatic) interaction between halogenated tubercidin ligands with Haspin. Consistent with previous theoretical finding, the site specificity of the XB binding over the *ortho*-carbon is identified in all cases. A systematic increase of the interaction energy down Group 17, based on both quantum chemical and MD results, supports the important role of halogen bonding in this series of inhibitors. The observed trend is consistent with the experimental observation of the trend of activity within the halogenated tubercidin ligands (F < Cl < Br < I). Furthermore, non-covalent interaction (NCI) plots show that cooperative non-covalent interactions, namely, hydrogen and halogen bonds, contribute to the binding of tubercidin ligands toward Haspin. The understanding of the role of halogen bonding interaction in the ligand–protein complexes may shed light on rational design of potent ligands in the future.

## 1. Introduction

**Figure 1:**
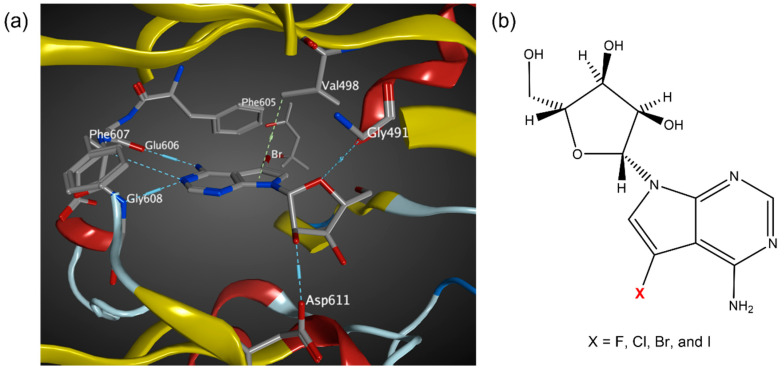
(**a**) Interaction site of tubercidin inhibitors with Haspin key residues (PDB 6G35). (**b**) Molecular structures of halogenated tubercidin derivatives.

Haspin, an atypical serine/threonine protein kinase, phosphorylates histone H3 at ‘Thr-3′ during mitosis [1,2,3]. The mRNA of Haspin was first detected and named cell-specific gene 2 (GSG2) in 1994, which was subsequently renamed as haploid germ cell-specific nuclear protein kinase (Haspin) [4]. Significantly different from other protein kinases, phosphorylation is not required for Haspin to be activated. During mitosis, a docking site is created for chromosome passenger complex (CPC), which plays a crucial role to prevent chromosome misalignment [5,6,7,8,9,10,11]. In addition to that, it is also involved in centromeric cohesion and mitotic spindle stability, making it a potential target for cancer therapy [12,13,14,15]. Human Haspin consists of 798 amino acids. The N-terminal part is a less conserved regulatory domain, while the C-terminal is a well-conserved catalytic kinase domain [16,17]. Recently, Chaikuad, Knapp and co-workers [18] identified 5-iodotubercidin (5-iTU), an adenosine derivative, as a potent Haspin inhibitor in vitro, showing the self-renewal and differentiation effect of mouse embryonic stem cells (ESCs) [19,20,21]. Figure 1a [18] shows the noncovalent interactions between tubercidin and Haspin key residues, including Gly491, Val498, Phe605, Glu606, Phe607, Gly608 and Asp611. 5-iTU inhibits Haspin with an IC_50_ ranged between 5 and9 nM [18,22,23,24]. The authors identified that potency and slow dissociation were increased with the increasing halogen size of 5-iTU derivatives (F < Cl < Br < I) (Figure 1b), which suggests the presence of the halogen bonding, specifically the halogen–aromatic π interactions.

Halogen bonding (XB) [25], driven by anisotropic charge distributions along the extension of the C–X bonds, is quantum-chemical in origin, with an equatorial ring of negative charge and a hind region of positive charge, termed as the σ-hole [26,27]. Halogens are shown to be useful to optimize ADMET properties and prolong the lifetime of the drug [28,29]. For protein–ligand interactions, halogen bonds can be formed between a halogenated ligand and accessible side chain groups, such as hydroxyls, carboxylates, sulfurs, nitrogen and π systems [30,31,32].

Based on an updated search on the Cambridge Crystallographic Database (CSD), we showed that π-type XB represents the majority (66%) of XB contacts identified [33]. For the aromatic type of XB acceptors, we observed a strong preference (95%) of XB binding towards the rim (i.e., over-bond or over-atom) of an aromatic ring, not towards the centroid. Quantum chemical studies of Cl_2_ and Br_2_ XB complexes of 14 polycyclic aromatic compounds, including aromatic amino acids, confirmed the observed rim specificity [33]. Intriguingly, the site specificity of the XB binding sites is identified in all cases. The authors demonstrated that the simple frontier orbital interaction readily rationalizes the rim and site specificities of XB involving aromatic XB-acceptors. In particular, the molecular orbital theory provides a proper description of the important charge transfer contribution in XB formation, supported by various energy decomposition analyses [33,34]. These theoretical studies demonstrate the prevalent role of π-type (particularly aromatic-π) XB acceptors in halogen bonded systems.

Non-covalent interactions play an important role in drug design. As one of the key noncovalent interactions, halogen bonding could contribute significantly to the ligand binding affinity and biological properties. In the present paper, we employed computational chemistry methods, both molecular dynamics (MD) simulations and quantum chemical calculations, to study the ligand–protein interaction between halogenated tubercidin derivatives and Haspin. Specifically, we investigated the role of the halogen aromatic π bond in the drug–protein binding.

## 2. Computational Methods

In this paper, Molecular Operating Environment (MOE) software and Amber14:EHT force field were used for molecular dynamics simulations [35,36]. The Amber14:EHT force field, which is an all-atomic combination of Extended Hückel Theory, is parameterized for non-bonded interactions. To benchmark the performance of the Amber14:EHT force field in prediction of halogen bonding in protein–ligand complexes, thyroid hormone receptor with ligands were simulated. The Berendsen thermostat was applied to control the simulation temperature [37]. The protocol of this MD simulation was started with 100 ps heat from 10 K to 300 K, followed by 1000 ps of NVT and 1000 ps NPT, at 300 K and 100 kPa, followed by a 100 ps of equilibration under constant temperature (300 K). The production was 8 ns at 300 K and 100 kPa, with a time step of 0.002 fs.

For the MD simulations of halogenated tubercidin ligands with a Haspin receptor, the native all-atomic crystal structures, namely, PDB 6G34 (5-iTU), 6G35 (5-brTU), 6G36 (5-clTU) and 6G37 (5-fTU), were taken as starting geometries. The geometries were optimized before proceeding to MD simulation. Hydrogen atoms were added, and partial charges were calculated based on the AMBER14:EHT forcefield. The partial charge for each atom is stored in the MOE internal atom data structure. Energy minimization was carried out using a succession of three algorithms, namely, steepest descent, conjugate gradient and truncated Newton. The protocol of simulation is the same as those of the benchmarking cases. However, the production run was longer at 25 ns at 300 K and 100 kPa, with a time step of 0.004 fs. Binding of the free energy of ligand–receptor complex was calculated using GBVI/WSA method [38] in MOE.

The counterpoise-corrected interaction energies of modeled halogenated ligands-receptor complexes were calculated using density functional theory (DFT) calculations based on the ωB97X-D functional [39]. The Haspin receptor was truncated to the key residue Phe605 only and the halogenated tubercidin ligands were modeled by halogenated pyrrolo[2,3-d]pyrimidine. The aug-cc-pVTZ basis set is used for non-iodine atoms and Def2-TZVPD basis set for iodine atom. It is important to note the ωB97X-D functional benchmarks well against high-level CCSD(T) method in binding energies of XB complexes involving aromatic acceptors [33,40]. Solvent effect was incorporated using the polarizable continuum model [41] to account for the non-specific (macroscopic) effect of the protein dielectric medium. A dielectric constant (ε) of 6 was used as the average dielectric constant inside protein is relatively low (about 6−7) [42,43]. Visualization of noncovalent interactions in ligand–receptor complexes was carried out using the NCI plot [44,45]. The NCI isosurfaces were visualized with VMD program [46] using data produced by Multiwfn program [47]. The strength of the noncovalent interaction is indicated by the color of the isosurface: green represents attractive while blue denotes strongly attractive. The NCI analysis has been successfully used to shed light on the presence of halogen bonding in various chemical systems [48,49,50,51].

## 3. Results and Discussion

### 3.1. Benchmark of AMBER14EHT Force Field on XB Ligand–Protein Complexes

**Figure 2:**
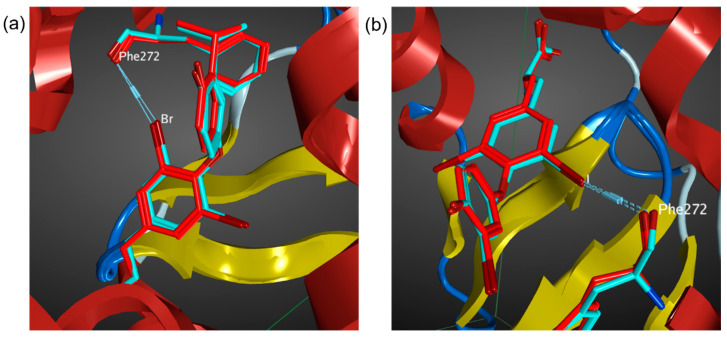
(**a**) Superimpose of MD predicted binding pose (blue) with crystal structure 2J4A (red). (**b**) Superimpose of MD predicted binding pose (blue) with crystal structure 1XZX (Red).

The performance of Molecular Operating Environment (MOE) was initially benchmarked with the thyroid hormone receptor complexed with brominated and iodinated ligands, PDB 2J4A and 1XZX, respectively [52,53]. The halogen bonds were identified between halogen atoms (Br and I) and Phe272 carbonyl oxygen atoms in the ligand–protein complexes. The distances for Br∙∙∙O and I∙∙∙O XBs in the crystal structures are 3.28 Å and 3.23 Å and the corresponding XB angles for Br∙∙∙OC and I∙∙∙OC are 163.6° and 165.9°, respectively. The MD simulations reproduced the halogen bond interaction presented in the crystal structures, with XB distances of 3.54 Å and 3.37 Å, and angles of 158.9° and 162.5°, respectively, for the Br∙∙∙O and I∙∙∙O halogen bonds (Figure 2). However, the XB distances were underestimated slightly by 8% and 4%, for the Br and I systems, respectively. These differences will be used to correct the XB distances in the following MD simulations of the halogenated tubercidin ligands.

### 3.2. MD Simulations of Halogenated Tubercidin Ligands with Haspin

**Figure 3:**
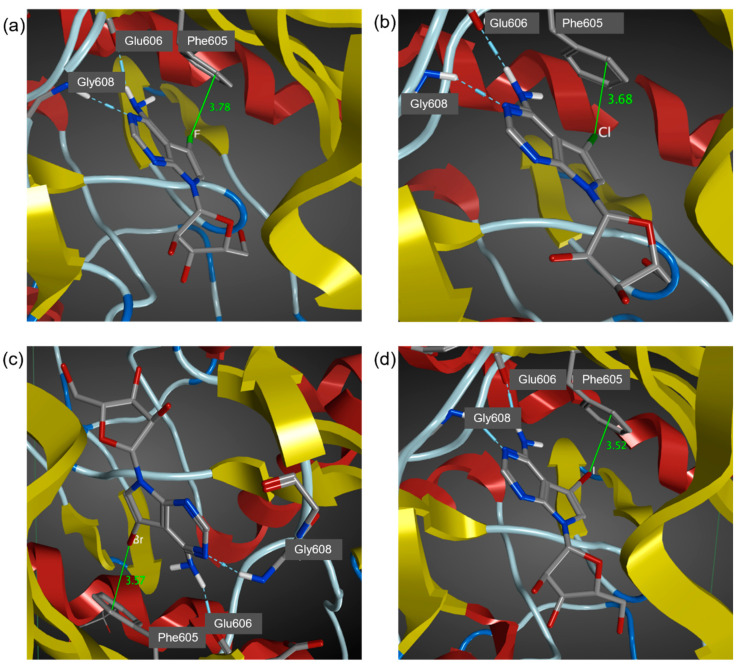
Key interaction sites of tubercidin inhibitors with Haspin from MD simulations. Interaction distance (Å) between halogen atom and C_α_ carbon of Phe605 phenyl ring: (**a**) 5-fTU, (**b**) 5-clTU, (**c**) 5-brTU and (**d**) 5-iTU. The geometries were chosen by the shortest distance from the last 50 snapshots for each MD trajectory.

Molecular dynamics simulation provides a dynamic model which can provide insight on ligand-receptor binding by providing information of internal motions and conformational change. In addition, the relative binding free energy predicted by MD simulation may provide a quantitative estimate of drug–protein interaction. The key halogen bond interaction is reproduced in the MD simulations based on the AMBER14:EHT force field (Figure 3), RMSD plot for 5-iTU-haspin MD simulation was provided in supporting information (Figure S1). Consistent with previous theoretical findings on rim and site specificity [33], the halogen∙∙∙π (aromatic) interaction between the halogen atom and the phenyl ring of gate keeper Phe605 is site specifically at the rim of the phenyl ring and is *ortho*-directed. The *ortho*-directed effect can be rationalized in terms of the charge polarization effect of a substituted alkyl group, such as toluene [33]. The X∙∙∙π distances were measure between the halogen atom and the closest carbon of the phenyl ring of Phe605 are 3.78, 3.68, 3.57 and 3.52 Å, for 5-fTU, 5-clTU, 5-brTU and 5-iTU, respectively (Figure 3). It was observed that the closest carbon atoms are always the *ortho* carbons on the aromatic ring of Phe605.

**Figure 4:**
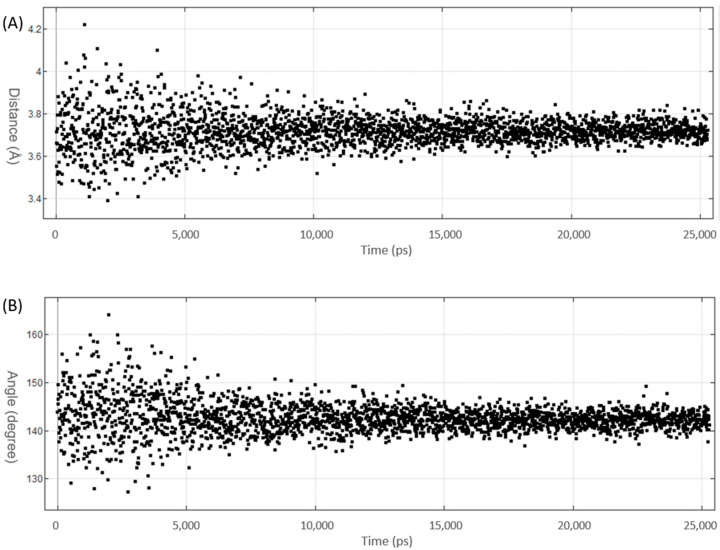
Plot of XB parameters of MD simulation trajectory of 5-iTU with Haspin receptor: (**A**) XB distance (I∙∙∙C_α_) and (**B**) XB angle (<C-I∙∙∙C_α_). The RMSD plot is given in Supporting Information (Figure S1).

On the basis of the benchmark MD simulations (Section 3.1), the XB distances are corrected for the significant underestimation. For the I, Br and Cl derivatives, the corrected X∙∙∙π interaction distance is less than the sum of van der Waal radii (Table 1). This indicates the presence of a halogen bond between the halogen atom and the aromatic ring (Phe605). In addition, trajectory plots of XB distance and angle (Figure 4) show that 5-iTU ligand stays tightly to the Haspin binding pocket throughout the simulation. This provides further evidence of a strong halogen bonding interaction between 5-iTU and the receptor. The estimated binding free energies, based on the GBVI/WSA_dG method are −46.1, −47.9, −48.6 and −48.9 kcal/mol, for F, Cl, Br and I derivatives, respectively. The GBVI/WSA_dG method is a forcefield based scoring function, which has been trained on 99 protein–ligand complexes dataset [54]. The values may be overestimated for ligand–protein complex, which do not fall in the dataset. However, the trend of binding energies of the halogenated tubercidin derivatives follows the trend of halogen bonding suggests the role of a halogen bond, specifically the halogen–aromatic π interaction. It is important to note that in addition to halogen bonds, various hydrogen bonds, e.g., between ligand NH and Glu606 carbonyl O atoms (see Figure 3), contribute to the overall binding energy listed in Table 1. Hence, it is instructive to perform DFT calculations (Section 3.3) to further assess the strength of halogen bonding quantitatively.

### 3.3. Quantum Chemical Calculations of Halogen Bond Interaction Energies

**Figure 5:**
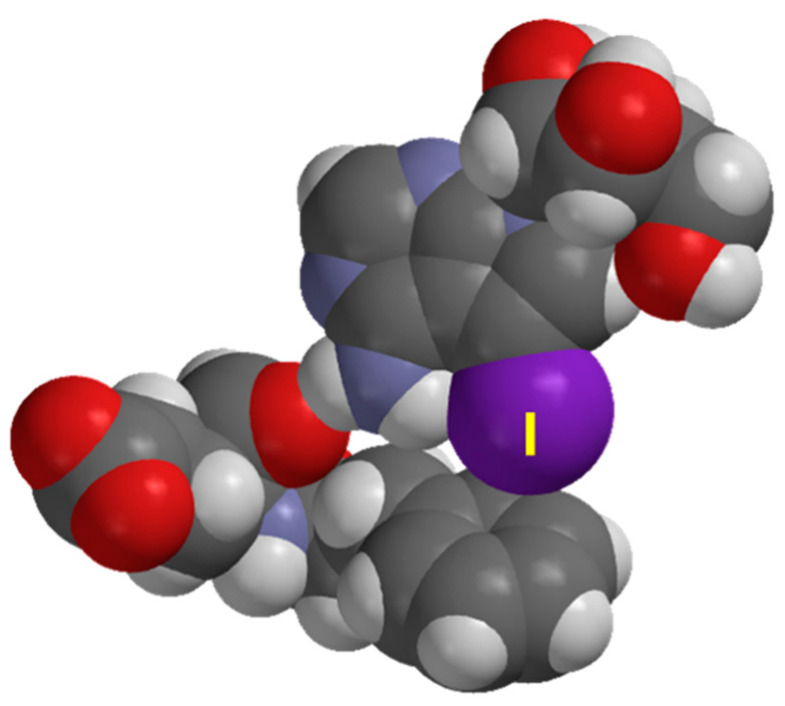
Space-filling model of close contact between 5-iTU and truncated receptor residues (Phe605 and Glu606) of PDB 6G34.

**Figure 6:**
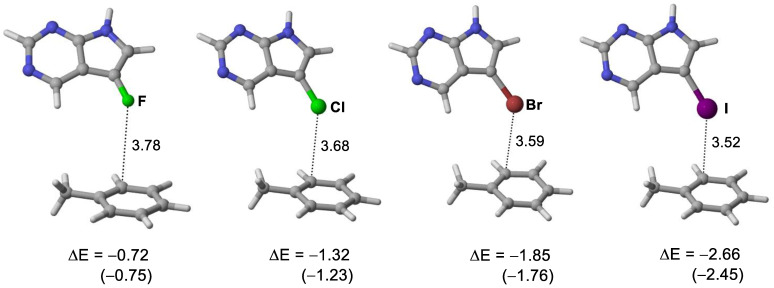
Structures and interaction energies (ΔE, kcal/mol) of modeled halogen bonded complexes. Calculated interaction energies in a dielectric medium of ε = 6 are given in parenthesis.

Careful examination of the X-ray structures of the ligand–receptor complexes reveals that the XB binding site is over the carbon atom of phenyl ring, not the centroid. Figure 5 depicts the space-filling model of the complex between XB donor 5-iTU and the aromatic side chain Phe605 and the adjacent Glu606 residue, which clearly indicates that over-atom binding mode at C_α_ (Phe605). The shortest halogen(X)–π distances over C_α_ of the phenyl ring in the crystal structures are 3.68, 3.59 and 3.52 Å for 5-clTU, 5-brTU and 5-iTU, respectively (Figure 6). The X∙∙∙π interaction distance is less than the sum of van der Waal radii for Br and I derivatives. The rim and site specificity of the X∙∙∙π (aromatic) interaction of the ligand–receptor complexes are readily confirmed by the MD simulations (Section 3.2).

To further shed light on the role of halogen bonding in this series of halogenated tubercidin ligands, DFT calculations were performed on truncated modeled systems of the four halogenated tubercidin derivatives (Figure 6) to quantify the interaction energy (DE) between the halogen containing moiety (XB donor) and the aromatic XB acceptor. In these modeled systems, only Phe605 key residue was included in the receptor and the halogenated tubercidin ligands were modeled by halogenated pyrrolo[2,3-d]pyrimidine. The moiety containing the NH_2_ group of tubercidin ligand was not included so that the hydrogen bond interaction is excluded in the interaction energy calculation. The modeled structures (see Figure 6) were derived from the crystal structures (6G34, 6G35, 6G36 and 6G37) without further optimization. We observed a systematic increase in the interaction energy (in vacuo) down Group 17, F (−0.72) < Cl (−1.32) < Br (−1.85) < I (−2.66 kJ/mol) (Figure 6). To stimulate the protein environment, implicit PCM solvation model was employed to examine the dielectric effect (ε = 6). The predicted medium effect on the interaction energies is small (~0.1 kcal/mol) and the trend of interaction energy remains the same (Figure 6). These trends of interaction energies readily support the importance role of halogen bonding in this series of inhibitors. The observed trend is consistent with the experimental observation of the trend of activity within the halogenated tubercidin ligands [18].

### 3.4. Analysis of Noncovalent Interactions

**Figure 7:**
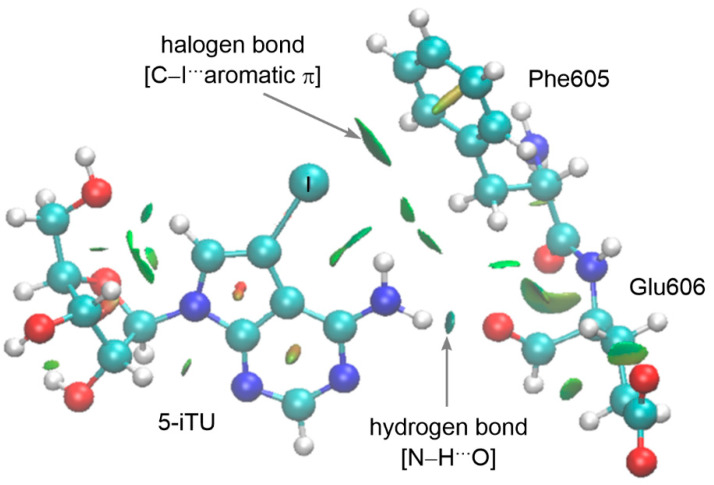
NCI isosurface between the tubercidin ligand 5-iTU and Haspin key residues (Phe605-Glu606) of PDB 6G34. Interaction strength increases from green to blue.

Finally, we employed the noncovalent interaction (NCI) plots to visualize the key noncovalent interactions between 5-iTU and the Haspin key residues. The noncovalent interactions index enables the visualization of noncovalent interactions through the transformation of the reduced electron density gradient into the surface, in which the color is representative of the nature (repulsive or attractive) and strength of the NCI [44,45]. The NCl plot of 5-iTU (Figure 7) reveals two types of attractive intermolecular NCIs, namely, the hydrogen and halogen bonds. The dark blue surface between the N–H proton of 5-iTU and the carbonyl oxygen of Glu606 moiety demonstrates the stronger N–H···O hydrogen bond. The presence of halogen bond is readily reflected in the green disc between the iodine atom of 5-iTU and the phenyl ring of Phe605. The existence of XB in the Br and Cl analogues and the non-existence of XB in the F analogue were also confirmed in the NCI analysis (see Supporting Information, Figure S2). In summary, cooperative noncovalent interactions, namely, hydrogen and halogen bonds, occur in the interaction between 5-iTU and Phe605-Glu606.

## 4. Conclusions

Discovered two decades ago, the atypical kinase Haspin plays an essential role in Histone H3 phosphorylation and as well as in CPC activity regulation, making itself an attractive target for cancer therapy. On the basis of QC calculations and MD simulations, we have confirmed the presence of halogen bonding between halogenated tubercidin ligands (Cl, Br and I) with the Haspin receptor. Site specificity of the halogen∙∙∙π (aromatic) interaction over the *ortho*-carbon (Phe605) is observed in the MD simulations. The trend of DFT calculated interaction energy (F < Cl < Br < I) supports the important role of halogen bonding in this series of halogenated inhibitors. With the understanding of the role of halogen bonding interaction, we hope this type of non-covalent interaction can be further exploited in the rational design of therapeutic drugs in the future.

## Figures and Tables

**Table 1:** Halogen bond distances and binding energies of halogenated tubercidin ligands with Haspin receptor obtained from MD simulations.

Ligands	X∙∙∙π Closest Carbon Distance (Å) ^a^	Sum of VDW Radii (Å) ^b^	Binding Energy (kcal/moL) ^c^
5-iTu	3.52 (3.38)	3.68	−48.9
5-brTu	3.57 (3.28)	3.55	−48.6
5-clTu	3.68 (3.39)	3.45	−47.9
5-fTu	3.78 (3.48)	3.17	−46.1

(^a^) Corrected XB distances, in parenthesis, based on benchmark comparison. (^b^) Van der Waals radii of C, F, Cl, Br and I are 1.70, 1.47, 1.75, 1.85 and 1.98 Å, respectively. (^c^) Binding free energy derived from molecular dynamic simulations.

## Supplementary Materials

The following supporting information can be downloaded, Figure S1. Root mean square deviation (RMSD) plot of complex between 5-iTU and Haspin; Figure S2. NCI isosurface between the tubercidin ligand 5-XTU and Haspin key residues (Phe605-Glu606): (a) 5-brTU, (b) 5-clTU, and (c) 5-fTU.

## Sample Availability

No sample is available from the authors.
