# Supplementary material for: Halogen Bonding in Haspin-Halogenated Tubercidin Complexes: Molecular Dynamics and Quantum Chemical Calculations
Source: Molecules. 2022 Jan 21;27(3):706. doi: 10.3390/molecules27030706 (PMC8840108; doi:10.3390/molecules27030706)
Supplement: Supplementary file 1 [file molecules-27-00706-s001.zip › molecules-1546141-supplementary.pdf]

Halogen Bonding in Haspin-Halogenated Tubercidin Complexes: Molecular Dynamics and Quantum Chemical Calculations

Yujing Zhou and Ming Wah Wong *

Department of Chemistry, National University of Singapore, 3 Science Drive 3, Singapore 117543

Email: chmwmw@nus.edu.sg

Supporting Information

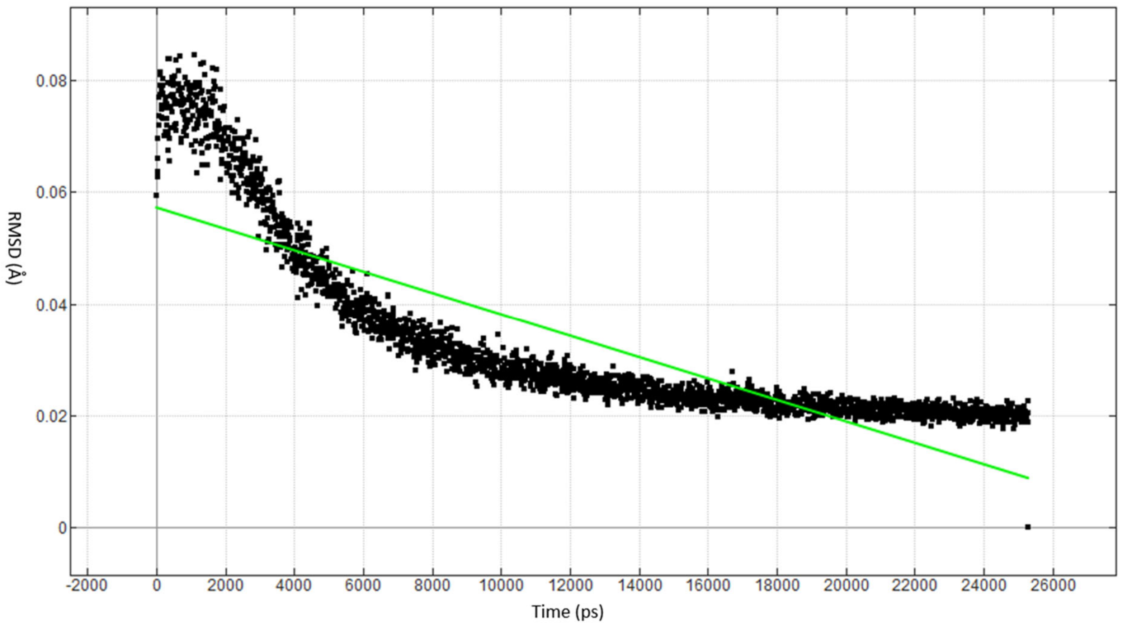

Figure S1. Root mean square deviation (RMSD) plot of complex between 5-iTU and Haspin.

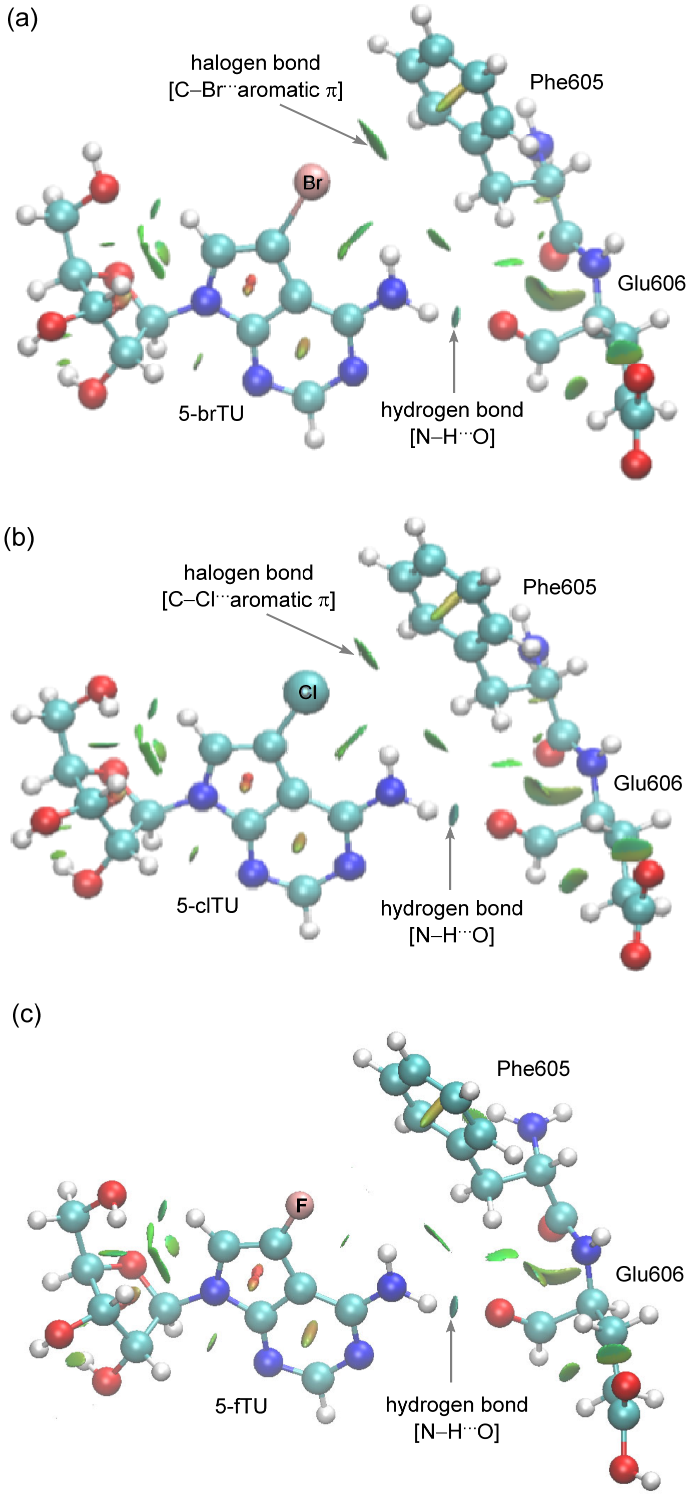

Figure S2. NCI isosurface between the tubercidin ligand 5-XTU and Haspin key residues (Phe605-Glu606): (a) 5-brTU, (b) 5-clTU, and (c) 5-fTU.
